# Superior Textured Film and Process Tolerance Enabled by Intermediate‐State Engineering for High‐Efficiency Perovskite Solar Cells

**DOI:** 10.1002/advs.201903009

**Published:** 2020-01-20

**Authors:** Shubo Wang, Yiqi Chen, Ruiyi Li, Yibo Xu, Jiangshan Feng, Dong Yang, Ningyi Yuan, Wen‐Hua Zhang, Shengzhong (Frank) Liu, Jianning Ding

**Affiliations:** ^1^ School of Materials Science and Engineering Jiangsu Collaborative Innovation Center for Photovoltaic Science and Engineering Jiangsu Province Cultivation Base for State Key Laboratory of Photovoltaic Science and Technology Changzhou University Changzhou Jiangsu 213164 China; ^2^ Key Laboratory of Applied Surface and Colloid Chemistry Ministry of Education Shaanxi Key Laboratory for Advanced Energy Devices Shaanxi Engineering Lab for Advanced Energy Technology School of Materials Science and Engineering Shaanxi Normal University Shaanxi Xi'an 710119 China; ^3^ Sichuan Research Center of New Materials Institute of Chemical Materials China Academy of Engineering Physics Chengdu Sichuan 610200 China; ^4^ Institute of Intelligent Flexible Mechatronics Jiangsu University Jiangsu Zhenjiang 212013 China

**Keywords:** intermediate‐state, perovskite solar cells, process tolerance, textured film

## Abstract

As the power conversion efficiency (PCE) of perovskite solar cells (PSCs) is increased to as high over 25%, it becomes pre‐eminent to study a scalable process with wide processing window to fabricate large‐area uniform perovskite films with good light‐trapping performance. A stable and uniform intermediate‐state complex film is obtained by using tetramethylene sulfoxide (TMSO), which extends the annealing window to as long as 20 min, promotes the formation of a high‐quality perovskite film with larger grains (over 400 nm) and spontaneously forms the surface texture to result in an improved fill factor and open‐circuit voltage (*V*_oc_). Moreover, the superior surface texture significantly increases the long‐wavelength response, leading to an improved short‐circuit current density (*J*_sc_). As a result, the maximum PCE of 21.14% is achieved based on a simple planar cell structure without any interface passivation. Moreover, a large area module with active area of 6.75 cm^2^ is assembled using the optimized TMSO process, showing efficiency as high as 16.57%. The study paves the way to the rational design of highly efficient PSCs for potential scaled‐up production.

## Graphical abstract

A stable intermediate‐state film is obtained by using teramethylene sulfoxide (TMSO), originating from the formation of stronger coordination bond between TMSO and all perovskite precursors, which extends the annealing window and promotes the formation of a high‐quality film with larger grains and textured surface. 21.14% efficiency is achieved attributable to the improvement of the long‐wavelength response and fill factor.

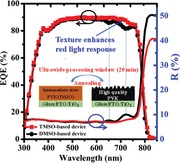

Perovskite solar cells (PSCs) have attracted intense research interest due to their unique properties, including low cost, large‐scale processability, and high efficiency. In the past several years, the PSC field has witnessed a remarkable increase in power conversion efficiency (PCE) from 3.8% to over 25%,^1^ which was comparable to the most established decades‐old commercial photovoltaic technologies. To date, most of the highly efficient PSCs were prepared by the solution‐chemistry approach.^2^, ^3^, ^4^, ^5^, ^6^, ^7^ However, for scaled‐up production, many issues, such as the wide processing window, light trapping (which has been widely used in traditional thin‐film solar cells), and fabrication of scaled‐up perovskite film with high‐quality, remain to be solved.

The “annealing window” (the storage time of the intermediate‐state films before annealing), was reported to be crucial for the formamidinium (FA)‐based perovskite system during the formation of a perovskite film prepared by the solution approach.^8^, ^9^, ^10^ In particular, high‐quality perovskite film was required for the highly efficient perovskite device, which could only be obtained when the thermal annealing of the perovskite intermediate state films was performed within the annealing window. The residual of the unwanted secondary phases was easily formed when the films were annealed outside of the window. Therefore, if the annealing window can be effectively expanded, the compatibility of the perovskite film preparation will be greatly improved, which is very beneficial for mass production.

On the other hand, the main contribution of the photocurrent density from the light response for solar cells originates from the long wavelengths including red‐ and near‐infrared regions. However, there were one or two valleys within 650–800 nm of the external quantum efficiency (EQE) spectra for PSCs,^2^, ^3^, ^4^, ^5^, ^6^, ^7^, ^11^, ^12^, ^13^, ^14^ which should be ascribed to the light reflection depending on the thickness and morphology of the film. Generally, a texture back reflector layer was introduced into thin‐film solar cells to further enhance the long‐wavelength response.^15^, ^16^

Herein, we simultaneously obtained high‐quality perovskite films with ultrawide annealing windows and spontaneous growth of the surface texture by using tetramethylene sulfoxide (TMSO) as a Lewis base ligand in perovskite precursor solution. Ligand is generally used to improve the quality of the perovskite film.^17^, ^18^, ^19^, ^20^, ^21^, ^22^, ^23^ Compared to general ligands, such as dimethyl sulfoxide (DMSO) and *N*‐methyl‐2‐pyrrolidone, TMSO can form a strong coordinate bonding with all perovskite precursors (PbI_2_, PbBr_2_, formamidinium iodide (FAI), methylammonium bromide (MABr), and CsI), which resulted in a stable and uniform intermediate‐state complex film after removing solvent (*N*,*N*‐dimethylformamide, DMF), restrained the spontaneous phase transition and additional nucleation, and extended the annealing window to as long as 20 min. In addition, the stable intermediate state changed the crystallization process and promoted the growth of larger gains and the formation of a surface texture. The valleys within 650–800 nm of the EQE spectrum were eliminated due to the spontaneously formed surface texture, which enhanced the photocurrent density. An impressive maximum PCE of 21.14% was achieved for the device with a simple planar structure (FTO/TiO_2_/perovskite/spiro‐OMeTAD/Au) without any surface passivation. The ultrawide processing window, high‐quality film, and back surface texture of this approach demonstrate its great potential for scaled‐up production on PSCs.

Two different solvent groups were used to prepare the FA‐based perovskite films: DMF:DMSO and DMF:TMSO. The composition of FA‐based perovskite was Cs_0.1_FA_0.85_MA_0.05_PbI_2.85_Br_0.15_. Here, the DMF is only used as a solvent due to the weak interaction with perovskite precursor. These solution were deposited on TiO_2_/FTO substrate by spin‐coating, the spin‐coated films were treated by vacuum quenching,^24^ and then the films transformed into perovskite intermediate‐state films (Figure S1, Supporting Information). The detailed process of device preparation is shown in the Supporting Information.

First, the annealing window for perovskite films derived from different ligands were investigated using X‐ray diffraction (XRD). As shown in **Figure** **1**a,b, for the annealed TMSO‐based films, all the diffraction peaks can be identified as the pure black phase (α‐phase) perovskite even after 20 min of storage (the storage time of the intermediate‐state films before annealing), No secondary phases (hexagonal polytypes) were detected,^25^ which indicates the formation of phase‐pure perovskite films. By contrast, for the annealed DMSO‐based films, secondary perovskite phases obviously appeared only after 2 min of storage, which is consistent with the reported results.^8^ This result is related to the nature of the intermediate‐state film. The XRD patterns of the intermediate‐state films with different ligands are shown in Figure 1c. Both the yellow phase (δ‐phase) and black phase (α‐phase) existed in the DMSO intermediate‐state film, only a weak peak corresponding to the δ‐phase can be detected for the TMSO‐based sample. Moreover, the diffraction peak for the TMSO‐based sample had a much lower intensity than the DMSO‐based sample, which indicates that the formation of perovskite nuclei (δ‐phase) were suppressed during vacuum quenching, on the contrary, for DMSO‐based sample, the lots of δ‐phase perovskite nuclei existed in the DMSO intermediate‐state film, which could spontaneously transform into secondary phase.^8^ Similar phenomenon can be observed for the TMSO‐ and DMSO–FAI–PbI_2_ intermediate‐state films (Figure S2, Supporting Information). In addition, Figure S3 of the Supporting Information shows that the color of the TMSO intermediate‐state film slightly changed after 20 min of storage, while the color of the DMSO‐based sample became dark brown after only 2 min, which is corresponding with the annealing window. Those results also indicate that TMSO could maintain stabilization of the intermediate‐state film, whereas DMSO intermediate‐state film was unstable because of additional nucleation and phase transition.^8^

**Figure 1:**
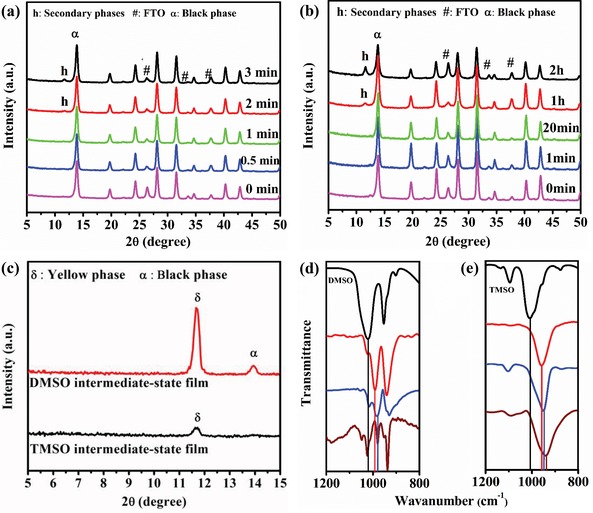
XRD patterns of the a) annealed DMSO‐ and b) TMSO‐based perovskite films with different storage times. c) XRD patterns of the intermediate‐state films based on TMSO and DMSO. FTIR spectra of the fingerprint regions for S=O stretching measured from d) liquid DMSO (black), DMSO–PbBr_2_ film (red), DMSO–PbI_2_ film (blue), DMSO intermediate‐state film (brown) and e) liquid TMSO (black), TMSO‐PbBr_2_ film (red), TMSO‐PbI_2_ film (blue), and TMSO intermediate‐state film (brown). The measurement of XRD and FTIR is performed on the fresh intermediate‐state films after a 1 min delay.

The stability of the intermediate‐state film without annealing was related to the formation of the complex between the ligands and the perovskite precursor.^17^, ^18^, ^19^, ^22^, ^26^ Figure 1d shows the Fourier transform infrared (FTIR) spectra for liquid DMSO, DMSO–PbI_2_ film, DMSO–PbBr_2_ film, and DMSO intermediate‐state film (the full spectra are shown in Figure S4a, Supporting Information). The stretching vibration peak of S=O (*v*(S=O)) appeared at ≈1020 cm^−1^ for liquid DMSO, which was shifted to 980 cm^−1^ (990 cm^−1^) for the DMSO–PbI_2_ film (DMSO–PbBr_2_ film), indicating the formation of DMSO–PbI_2_ (PbBr_2_) complexes.^21^ However, the *v*(S=O) of the DMSO intermediate‐state film hardly changed compared to the DMSO–PbI_2_ film, indicating that the coordinate interaction between DMSO and other perovskite precursors was relatively weak, therefore, DMSO‐based complexes was unstable and the DMSO molecule could spontaneously separate from the DMSO‐based complexes, thus, dissociative DMSO can be detected by FTIR for all DMSO‐based complex films (DMSO–PbI_2_ film, DMSO‐PbBr_2_ film, DMSO intermediate‐state film). In particular, for DMSO intermediate‐state film, the separation of DMSO leaded to additional nucleation. The FTIR spectra of the liquid TMSO, TMSO–PbBr_2_ film, TMSO–PbI_2_ film, and TMSO intermediate‐state film are shown in Figure 1e (the full spectra are shown in Figure S4b, Supporting Information). *v*(S=O) of liquid TMSO appeared at ≈1010 cm^−1^, which was shifted to 960 and 950 cm^−1^ for the TMSO–PbBr_2_ and TMSO–PbI_2_ film, respectively. The more intense low‐frequency shift (60 cm^−1^, (50 cm^−1^)) of *v*(S=O) for the TMSO–PbI_2_ (PbBr_2_) film (compared to liquid TMSO) displays the formation of stronger coordination bond between TMSO and PbI_2_ (PbBr_2_) than that of DMSO (40 cm^−1^ (30 cm^−1^)). In addition, the thermogravimetric analysis (TGA) spectra (Figure S5, Supporting Information) indicated that the decomposition temperature of TMSO–PbI_2_ complex powder was higher than that of the DMSO‐based sample, and the identical trend can be found on TMSO–PbBr_2_ complex powder. Furthermore, *v*(S=O) of the TMSO intermediate‐state film was reduced by 10 cm^−1^ compared to the TMSO–PbI_2_ films, which can be ascribed to the coordination reaction between TMSO and other perovskite precursor (CsI, MABr, FAI). These results can also be confirmed by the FTIR spectra of TMSO‐(FAI, CsI, MABr) and DMSO‐(FAI, CsI, MABr) solution, the *v*(S=O) of the TMSO‐(FAI, CsI, MABr) solution was reduced by 25 cm^−1^ compared to liquid TMSO, but hardly changed for that of DMSO (Figure S6, Supporting Information), correspondingly, the color of the TMSO‐(FAI, CsI, MABr) solution significantly changed compared to that of DMSO due to the formation of an electron donor–acceptor complex (Figure S7, Supporting Information),^18^ which implies that TMSO more strongly interacted with (FAI, CsI, MABr) than DMSO. In addition, no dissociative TMSO can be detected for any TMSO‐based sample (TMSO–PbI_2_ film, TMSO–PbBr_2_ film, and TMSO intermediate‐state film). In summary, compared to DMSO, TMSO can react with all perovskite precursors (in particular, the strength of the coordination bond for TMSO–PbI_2_(PbBr_2_) is more intense than that of DMSO) and form a more stable intermediate‐state films, which inhibits the reaction between organic salt and lead salt, therefore, the formation of the perovskite nuclei was suppressed during vacuum quenching,^22^, ^27^ and the additional nucleation for intermediate‐state film was delayed when storing. As a result, compared to DMSO, the TMSO can extend the annealing window from 2 min to as long as 20 min. It should be noted that, in order to guarantee the comparability between the two films in this study, 1 min delay time for the fresh intermediate‐state films before annealing was adopted for the TMSO‐ and DMSO‐based samples unless otherwise stated.

**Figure** **2**a,b shows the 45°‐viewing SEM images for the perovskite films based on TMSO and DMSO ligands. The significantly increased crystal grains are clearly observed for the TMSO system compared to the DMSO‐based perovskite films. Moreover, the grain of the TMSO‐based sample grows across the complete film thickness, which is favorable for the charge transportation.^6^ By contrast, the DMSO‐based sample shows more grain boundaries within the layer thickness (Figure S8, Supporting Information). This increased grains should be originated from the process of nucleation and crystallization.^17^, ^22^, ^25^, ^26^, ^27^, ^28^, ^29^ As above, TMSO could form a stronger coordination bond with all perovskite precursors (PbI_2_, PbBr_2_, FAI, MABr, and CsI) compared to DMSO, and resulted in a more stable intermediate‐state, which implies a higher critical Gibbs free energy (Δ*G*_c_) that decreases the rate of nucleation according to the classical nucleation theory.^30^ Therefore, there were few δ‐phase nuclei in the TMSO intermediate‐state film after vacuum quenching, by contrast, lots of δ‐phase nuclei existed in DMSO intermediate‐state film (Figure 1c). In addition, Figure 2c show that the process of crystal growth of TMSO sample was more longer than DMSO, and complex phase correspond to low‐angle diffraction peaks (≈7.5° and ≈9°) appeared during annealing, by contrast, no complex phase can be detected for DMSO sample (Figure S9, Supporting Information). Therefore, the lower nucleation density and longer process of crystallization lead to larger gain size of the perovskite film after the annealing.

**Figure 2:**
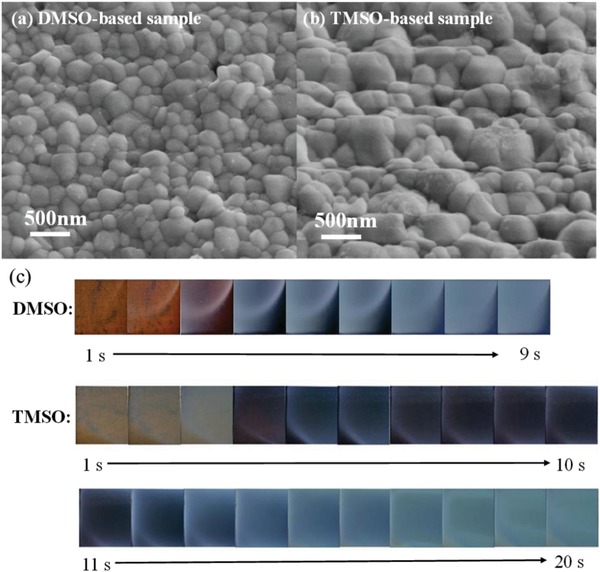
45°‐viewing SEM images for the a) DMSO‐ and b) TMSO‐based annealed perovskite films. c) Photos of the annealing process for the DMSO‐ and TMSO‐based perovskite films.

The larger grains result in less defect recombination.^31^, ^32^, ^33^ Photoluminescence (PL) and time‐resolved PL spectra (TRPL) were further applied to evaluate the quality of the perovskite films (the structure of samples are quartz glass/PMMA/perovskite/PMMA). The TMSO‐based sample had an obviously stronger PL intensity than the DMSO‐based sample (**Figure** **3**a). Correspondingly, the decay constants (for the radiative recombination of free charge carriers that originate from the bulk perovskite obtained by a double exponential fitting) increased from 0.69 to 1.87 µs for the perovskite films based on DMSO and TMSO (Figure 3b), which indicates that the quality of the TMSO‐based perovskite films was greatly improved.^34^, ^35^ The thermal stability of the two films are also studied via using the UV–vis absorption spectrum. Compared to the DMSO‐based film, there is no significant change in the absorption spectra for the TMSO‐based sample after 4 h annealing at 130 °C under 30% RH (Figure S10, Supporting Information).

**Figure 3:**
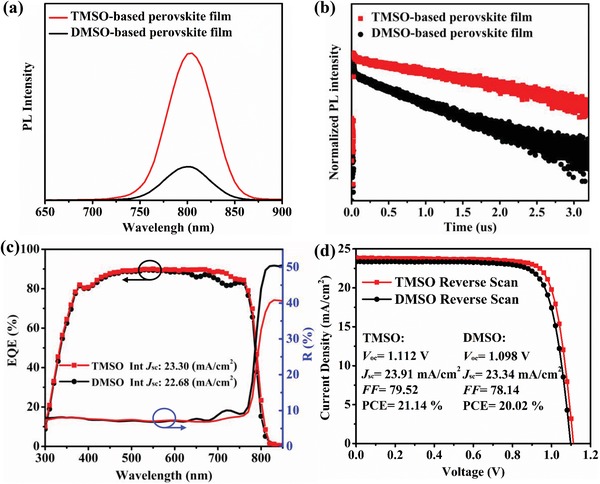
a) Steady PL and b) TRPL decay profiles (measured at the peak emission of 800 nm) for the annealed TMSO‐ and DMSO‐based perovskite films, the structure of the sample is quartz glass/PMMA/perovskite film/PMMA. c) EQE spectra and 8° angle integral total reflection for TMSO‐ and DMSO‐based PSCs. d) *J*–*V* curves of the best performing devices based on TMSO and DMSO measured under the standard AM 1.5 solar radiation with the reverse scan. The device area is 0.09 cm^2^.

It is worth mentioning the TMSO‐based film spontaneously formed a rougher surface after annealing because of high proportion (≈50%) of larger grains (over 400 nm) than DMSO‐based film, as shown in the 45°‐viewing (Figure 2a,b), the statistical distributions of the grain size for DMSO‐ and TMSO‐based perovskite film are shown in Figure S11 of the Supporting Information. The surface roughness (root mean square, RMS) of the films can be confirmed using the 3D profilometer, as demonstrated in Figure S12 of the Supporting Information. The RMS value of the TMSO‐based perovskite films is 111.8 nm, which is ≈2 times that of the DMSO sample (45.5 nm). Additionally, the textured film shows much lower reflectivity and higher light absorption compared to the DMSO‐based sample (Figure S13, Supporting Information), which is favorable for more effective light‐harvest for higher Jsc.^18^, ^36^ Similar light‐trapping strategy has been used in other types of thin‐film solar cells.

The D8 integral total reflection spectra of the complete solar cell are shown in Figure 3c. The reflection of 650–800 nm was significantly reduced in the TMSO device compared to the DMSO device because of the light‐trapping effect of the back surface texture. The EQE values exhibit a similar trend (Figure 3c), i.e., the two valleys within 650–800 nm disappeared for the TMSO device compared to the control device. The enhancement of the EQE value results in the increased integrated photocurrent density from 22.68 to 23.30 mA cm^−2^ (the absorption layer exhibits a similar thickness, Figure S8, Supporting Information). The current density–voltage (*J*–*V*) characteristics of our best performing cells are shown in Figure 3d. The short‐circuit current density (*J*_sc_), open‐circuit voltage (*V*_oc_), and fill factor (FF) under reverse scan were 23.91 mA cm^−2^, 1.112 V, and 79.52%, respectively, yield a PCE of 21.14% with negligible hysteresis (the reverse scan and forward scan data are shown in Figure S14, Supporting Information), whereas the PCE for the maximum control device was 20.02% with *J*_sc_ = 23.34 mA cm^−2^, *V*_oc_ = 1.098 V, and fill factor (FF) = 78.14%. Moreover, the present method shows excellent reproducibility, which can be confirmed from the statistical distribution of the photovoltaic performance (Figure S15, Supporting Information). The device stability was also monitored under 1 sun continuous illumination (30% RH) (Figure S16, Supporting Information). The unsealed TMSO‐based device maintains about 54% of its initial PCE after 96 h light aging, whereas the control device degrades by 84% under the same conditions. The improved device performance of the TMSO‐based solar cells can be attributed to following reasons: a) more stable intermediate‐state has been formed in the TMSO system lead to larger crystal grains and higher quality film, resulting in higher *V*_oc_ and FF; b) the TMSO system spontaneously formed a textured surface, which provides better light‐trapping effect. The reduced reflection leads to a significantly increased *J*_sc_. Based on these two factors, superior device performance has been achieved for the TMSO‐based device.

In addition, adopting a 20 min annealing window, the maximum PCE of TMSO‐based device is 21.01%, whereas the PCE for the DMSO‐base device is only 16.71% due to deterioration of film quality (Figure S17, Supporting Information). Meanwhile, a larger area module with active area of 6.75 cm^2^ has also been assembled based on TMSO. The module gives an efficiency of 16.57% (**Figure** **4**). Notably, the intermediate‐state film can be stored in glovebox for as long as 20 min before further processing without affecting module performance, demonstrating that TMSO can allow widened processing window which is desirable for scaled‐up production.

**Figure 4:**
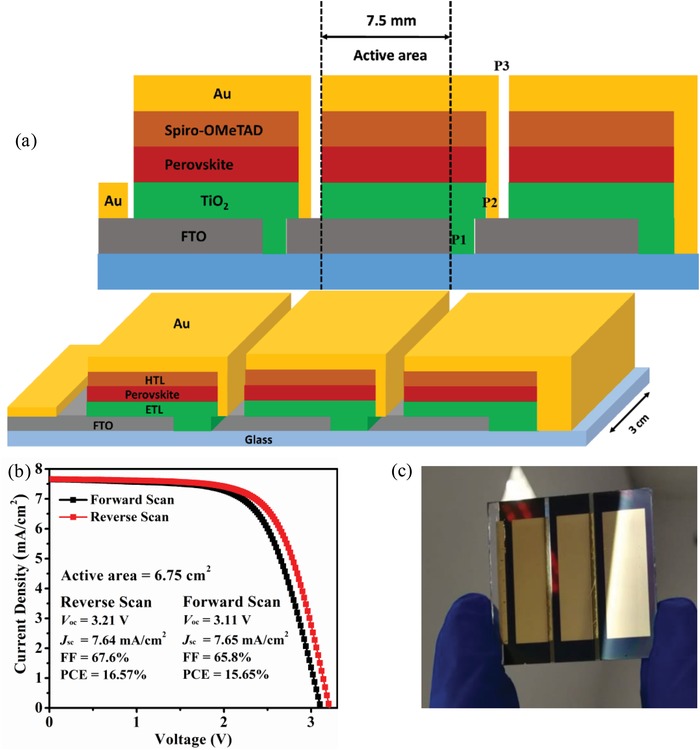
a) Schematic illustration of perovskite solar module structure, b) *J*–*V* curves of the best performing large‐area perovskite solar module under reverse scan (3.5 to −0.1 V) and forward scan (−0.1 to 3.5 V), the device active area is 6.75 cm^2^, and c) photo of a large‐area perovskite solar module.

In summary, a stable and uniform intermediate‐state complex film is obtained by using TMSO, which supplies the ultrawide processing window, a high‐quality film and a spontaneously formed surface texture, which is very beneficial for commercial applications and crucial for the improvement of the longwave response of PSCs. Our study paves the way to a rational design of highly efficient PSCs for future practical applications.

## Experimental Section

##### Materials

PbI_2_ (>98%), was purchased from TCI. PbBr_2_ (>98%) and CsI (>99.9%) were purchased from Sigma‐Aldrich. DMF (>99.9%), DMSO (>99.9%), TMSO (>99.9%), and NbCl_5_ (99.9%) were purchased from Alfa Aesar. FAI, MABr, and spiro‐OMeTAD were purchased from Xi'an Polymer Light Technology. TiCl_4_ (99.9%) was purchased from Aladdin. All the materials were used without purification. Fluorine‐doped tin oxide on glass (FTO glass, square resistance: 6–8 ohm sq^−1^, Haze: 11–17%) was obtained from Asahi Glass Co., LTD (AGC).

##### TMSO–PbI_2_ (PbBr_2_) and DMSO–PbI_2_ (PbBr_2_) Complex Films

0.5 mmol of PbBr_2_ and TMSO (DMSO) was dissolved in 800 µL of DMF, 1 mmol of PbI_2_ and TMSO (DMSO) was dissolved in 1 mL of DMF, those solution were deposited on quartz glass substrate by spin‐coating (4000 rpm for 10 s), the spin‐coated film was treated via vacuum quenching, and then, the film transformed into TMSO–PbI_2_ (PbBr_2_) or DMSO–PbI_2_ (PbBr_2_) complex films (the details of the processing can be found below). Those films were used for FTIR measurement.

##### TMSO–PbI_2_ (PbBr_2_) and DMSO–PbI_2_ (PbBr_2_) Complex Powders

1 mmol of PbI_2_ (PbBr_2_) was dissolved in 2 mL of TMSO (DMSO), anhydrous ethanol was added to the solution with drop by drop, the as‐prepared white precipitation was filtered and dried in vacuum for 6 h at room temperature, which was used for TGA measurement.

##### Device Fabrication

FTO substrates were ultrasonically cleaned with acetone, ethanol, and deionized water, respectively. Afterward, they were dried with nitrogen stream, and cleaned by UVO for 10 min immediately before using. The Nb doped TiO_2_ (Nb/Ti = 0.05, mol/mol) layer (30–40 nm) was prepared by the chemical bath deposition method (0.2 m TiCl4 aqueous solution with NbCl_5_ hydrochloric acid (38%) solution (0.18 g mL^−1^) at 70 °C for 76 min).

The Cs_0.1_FA_0.85_MA_0.05_PbI_2.85_Br_0.15_ precursor solution was prepared in 1.35 m PbX_2_ (X = I, Br) in the mixed solvent of DMF and TMSO (DMSO) with the molar ratios of PbX_2_/TMSO (DMSO) = 1:1. The solution was stirred at 50 °C for 3 h and filtered using a 0.22 µm PTFE filter element. The solution was spin‐coated onto the TiO2 substrates at 4000 rpm for 10 s. Then, the wet film was placed into a stainless‐steel chamber (diameter: 10 cm; height: 3 cm) connected to a rotary vane vacuum pump (vacuum capacity: 16 m^3^ h^−1^) and covered with quartz glass; the valve of the pump was subsequently opened, and the pressure of the chamber was rapidly dropped; when the color of the wet film changed, it transformed into intermediate‐state perovskite films. Then, the valve of the pump was closed and the chamber was rapidly purged. The films were stored in the glovebox (if necessary), the substrate was subsequently transferred to a hotplate and annealed at 130 °C for 15 min. All processes were in the glovebox, and the temperature of operation was 26 °C (Figure S1, Supporting Information).

The HTL was prepared by spin coating at 4000 rpm for 20 s using a chlorobenzene solution prepared by dissolving 90 mg spiro‐OMeTAD in 1 mL chlorobenzene with the addition of 45 µL Li‐TFSI/acetonitrile (170 mg mL^−1^) and 75 µL^−1^ KF209/acetonitrile (100 mg mL^−1^), and 10 µL TBP. Finally, 100 nm of gold was prepared by thermal evaporation.

##### Characterization

The XRD spectra of annealed perovskite films were obtained using a D/max 2500 PC X‐ray diffractometer with Cu, Kα radiation (Rigaku Corporation).The XRD spectra of intermediate‐state films were obtained using a DX‐2700 X‐ray diffractometer with a N_2_ flow near the samples (Haoyuan Instrument Co., Ltd.). The FTIR spectra were obtained using Nicolet iS10 (Thermo Scientific). The liquid samples were measured on KBr substrates, and the film samples were measured on quartz glass with the attenuated total reflection mode. Thermogravimetric analyses of the prepared TMSO (DMSO)–PbI_2_ and TMSO (DMSO)–PbBr_2_ complex powders were performed using HTG‐1 (Beijing Hengjiu Experimental Equipment Co. Ltd.) by 1 °C min^−1^ from room temperature to 300 °C under nitrogen atmosphere. The 45°‐viewing and cross‐sectional SEM images were obtained by using a field‐emission scanning electron microscope (S‐4800. Hitachi Corporation). The time‐resolved PL decay was measured with a DeltaFlex lifetime system (HORIBA, IBH, GLA). A picosecond pulsed laser with a wavelength of 505 nm was used; the repetition rate was set as 250 kHz for the time window of 3.2 µs. TRPL decay profiles were obtained at the peak emission (800 nm); parameter τ_2_ (corresponding to the radiative recombination from the bulk perovskite) was obtained via double exponential fitting. The RMS was measured using a Contour GT noncontact 3D profilometer (Bruker Corporation). The reflectivity, absorption, and transmittance spectra were measured using an UV–vis spectrometer (UV‐2600, Shimadzu Corporation). The *J*–*V* characteristics of photovoltaic cells were measured by a Keithley 2400 source and the solar simulator with standard AM 1.5 G (100 mW cm^−2^, SSF5‐3A: Enlitech). The light intensity was calibrated by applying a Si reference cell with a KG5‐filter (SRC‐2020‐KG5‐RTD, Enlitech), The *J*–*V* curves were measured by forward (−0.1 to 1.2 V forward bias) or reverse (1.2 to −0.1 V) scans; the step was 0.02 V; the delay time between steps was 50 ms. The EQE was measured using an Enlitech EQE measurement system (QE‐R3011, Enli Technology Co., Ltd.). An 8° angle integral total reflection of device was measured by the same system. A 3*3 mm metal mask was used for the EQE and *J*–*V* measurement.

## Conflict of Interest

The authors declare no conflict of interest.

## Supporting information

Supporting Information
