# Supplementary material for: Superior Textured Film and Process Tolerance Enabled by Intermediate‐State Engineering for High‐Efficiency Perovskite Solar Cells
Source: Adv Sci (Weinh). 2020 Jan 20;7(5):1903009. doi: 10.1002/advs.201903009 (PMC7055579; doi:10.1002/advs.201903009)
Supplement: Supplementary file 1 — Supporting Information [file ADVS-7-1903009-s001.pdf]

Copyright WILEY-VCH Verlag GmbH & Co. KGaA, 69469 Weinheim, Germany, 2020.

ADVANCED SCIENCE
Open Access

Supporting Information

for *Adv. Sci.,* DOI: 10.1002/advs.201903009

Superior Textured Film and Process Tolerance Enabled by Intermediate-State Engineering for High-Efficiency Perovskite Solar Cells

Shubo Wang, Yiqi Chen, Ruiyi Li, Yibo Xu, Jiangshan Feng, Dong Yang, Ningyi Yuan, Wen-Hua Zhang,* Shengzhong (Frank) Liu,* and Jianning Ding**

WILEY-VCH

Copyright WILEY-VCH Verlag GmbH & Co. KGaA, 69469 Weinheim, Germany, 2019.

Supporting Information

Superior Textured Film and Process Tolerance enabled by Intermediate-state Engineering for High-Efficiency Perovskite Solar Cells

Shubo Wang, Yiqi Chen, Ruiyi Li, Yibo Xu, Jiangshan Feng, Dong Yang, Ningyi Yuan, Wen-Hua Zhang*, Shengzhong(Frank) Liu*, Jianning Ding**

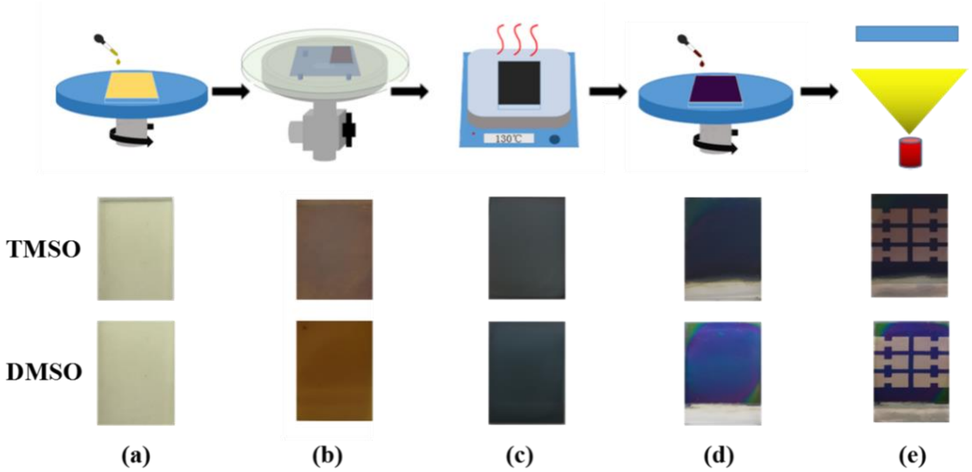

Figure S1. Schematic illustration of the preparation process of perovskite solar cells. (a) Spin-coating of the perovskite precursor solution (liquid film), (b) Vacuum quenching (intermediate-state perovskite film), (c) Annealing (black phase perovskite film), (d) Spin-coating of HTM, (e) Thermal evaporation of the Au electrode.

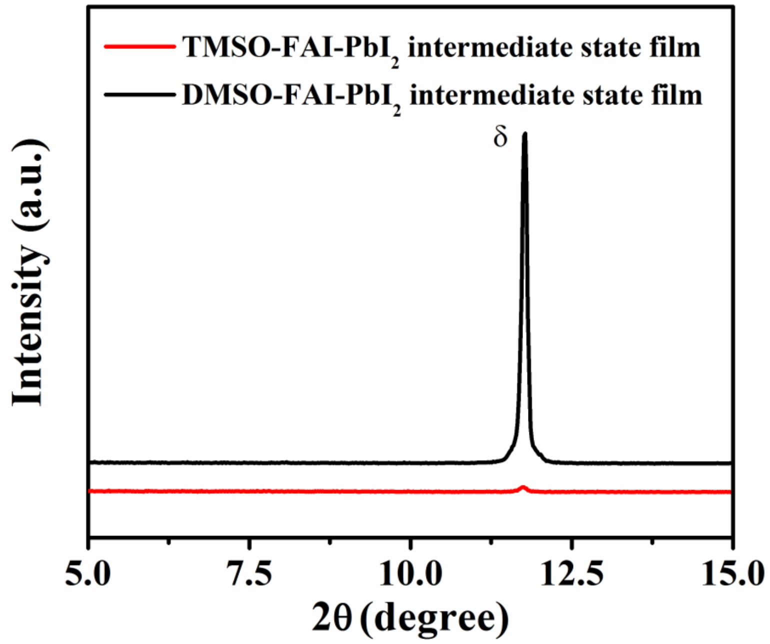

Figure S2. The XRD patterns of TMSO- and DMSO-FAI-PbI_2 intermediate-state film. The measurement is performed on the fresh intermediate-state films after a 1 min delay.

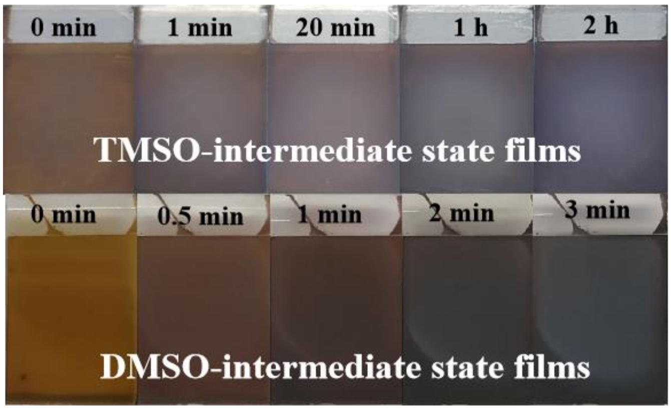

Figure S3. Photos of intermediate state films based on TMSO and DMSO with different storage times before annealing.

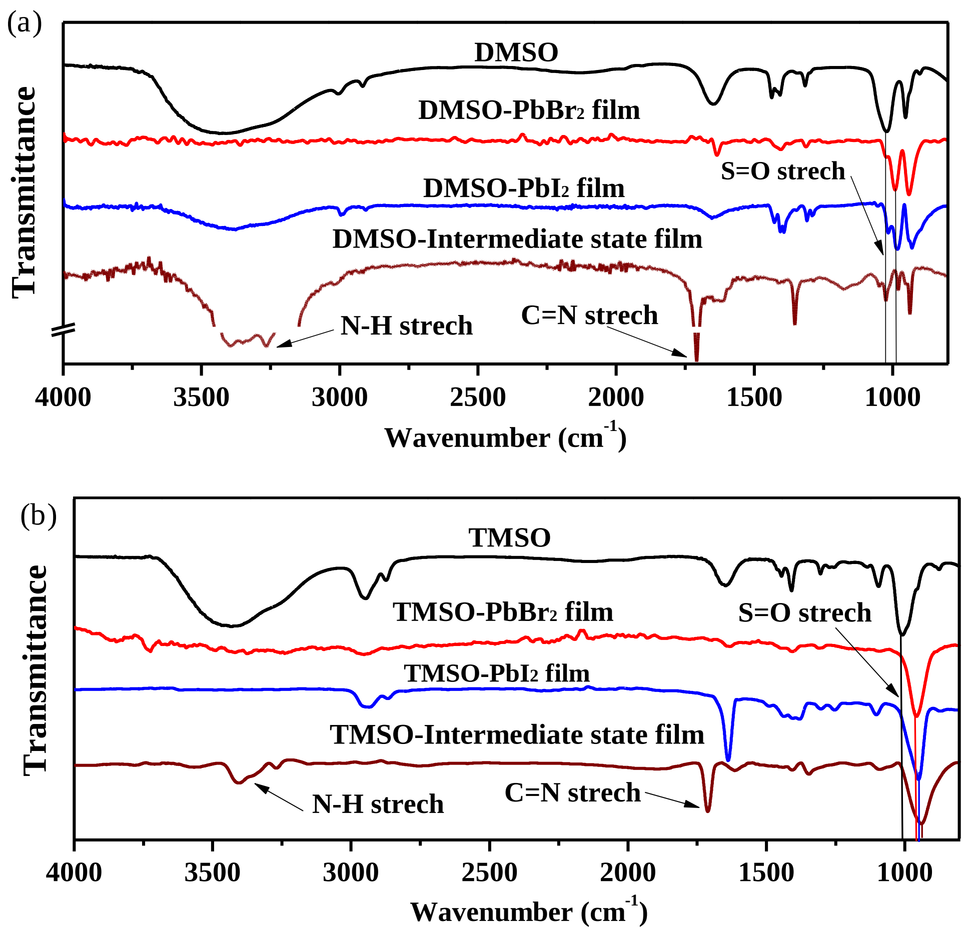

Figure S4. Fourier transform infrared (FTIR) spectra of the (a) liquid DMSO, DMSO-$PbBr_2$ film, DMSO-PbI_2 film, and DMSO intermediate-state film, (b) liquid TMSO, TMSO-$PbBr_2$ film, TMSO-PbI_2 film, and TMSO intermediate-state film. The measurement is performed on the fresh intermediate-state films after a 1 min delay.

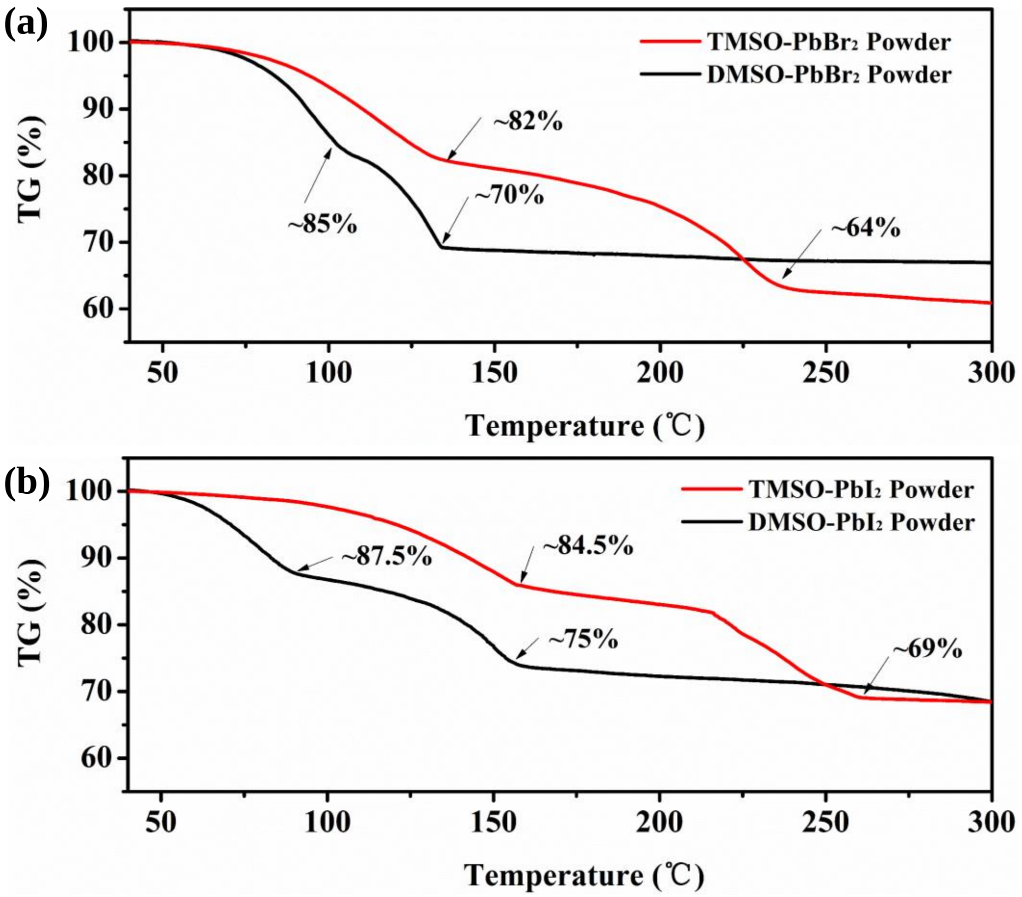

Figure S5. Thermogravimetric analysis (TGA) of (a) TMSO-$PbBr_2$ and DMSO-$PbBr_2$ powders, (b) TMSO-PbI_2 and DMSO-PbI_2 powders. The measurement was performed under an N_2 atmosphere with a heating rate of 1 °C/min.

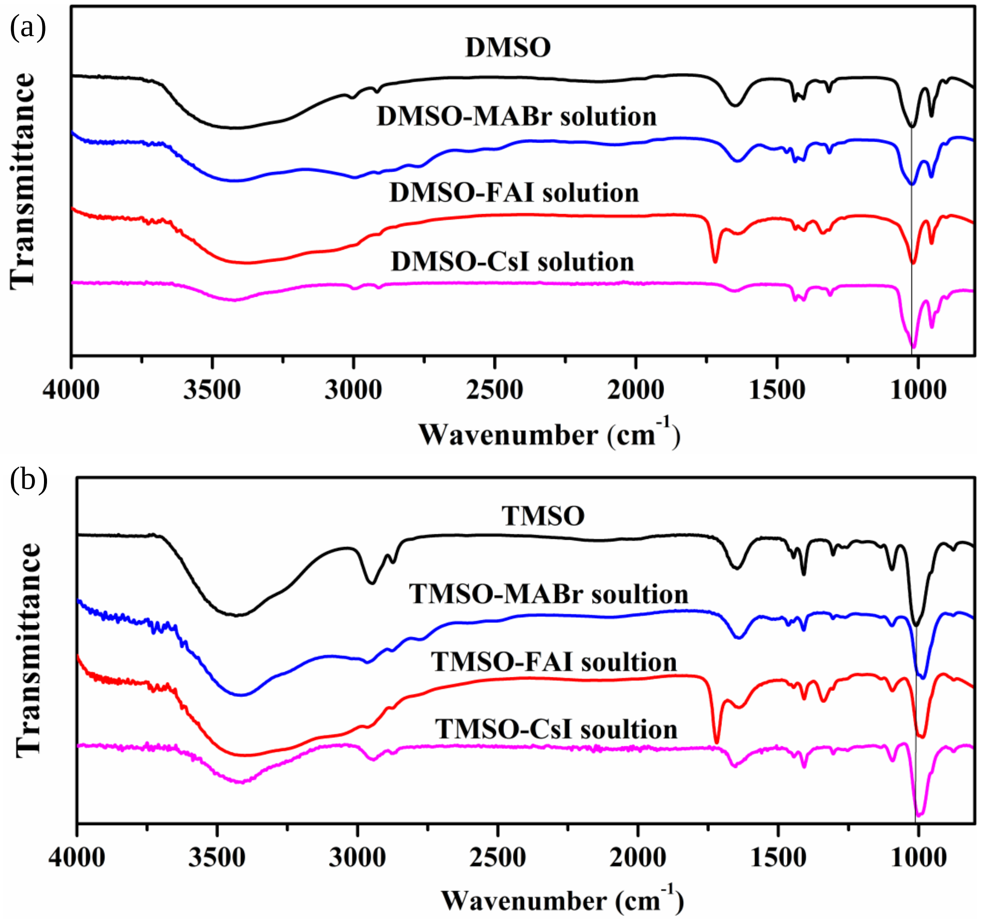

Figure S6. Fourier transform infrared (FTIR) spectra of (a) liquid DMSO, DMSO-MABr solution (3:1 mol/mol), DMSO-FAI solution (3:1 mol/mol), DMSO-CsI saturation solution, (b) Liquid TMSO, DMSO-MABr solution (3:1 mol/mol), TMSO-FAI solution (3:1 mol/mol), and TMSO-CsI saturation solution. All solutions were stirred at 50 °C for 3 h before measurement.

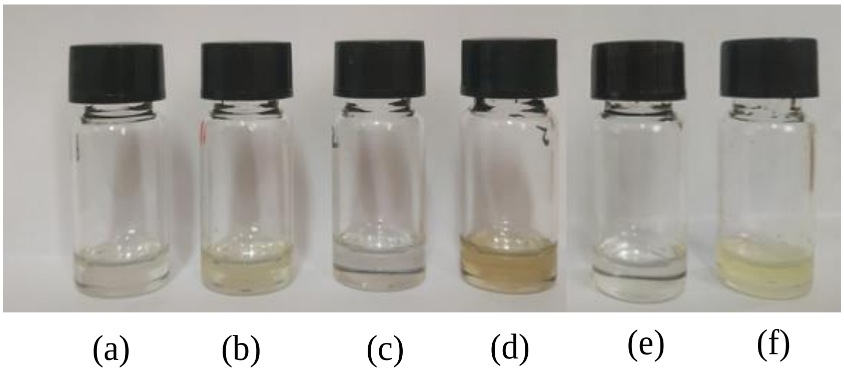

Figure S7. Photos of the (a) DMSO-FAI solution (3:1, mol/mol), (b) TMSO-FAI (3:1, mol/mol), (c) DMSO-MABr solution (3:1, mol/mol), (d) TMSO-MABr solution (3:1, mol/mol), (e) DMSO-CsI saturated solution, and (f) TMSO-CsI saturated solution. All solutions were stirred at 50 °C for 3 h.

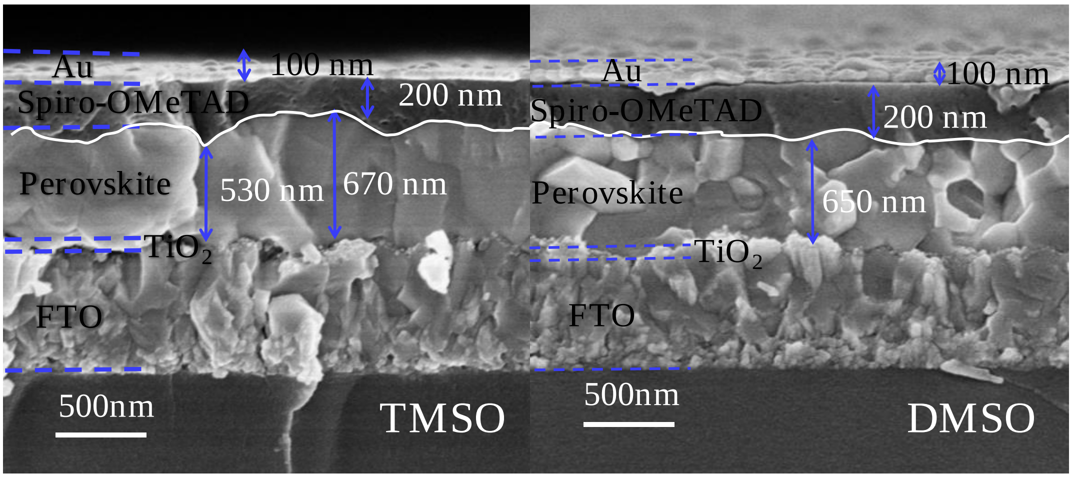

Figure S8. Cross-sectional SEM image of complete (a)TMSO- and (b)DMSO-based device.

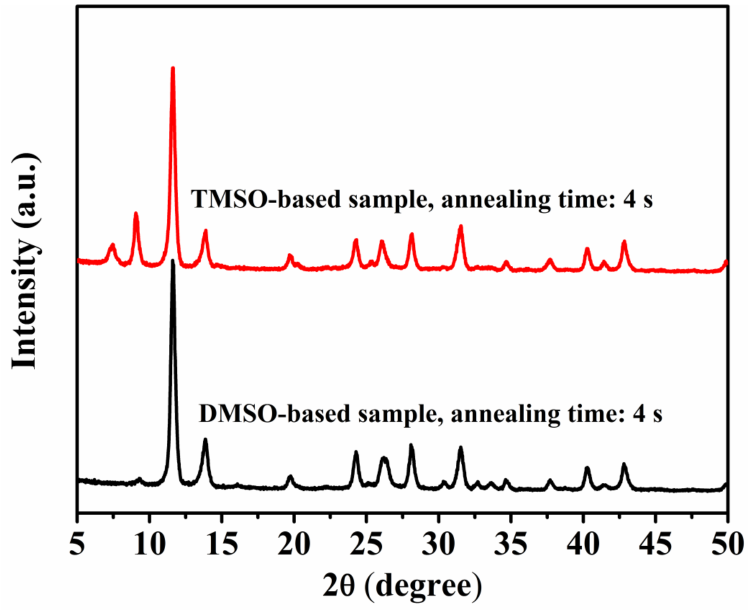

Figure S9. XRD pattern of the TMSO- and DMSO-based perovskite films with 4 s annealing.

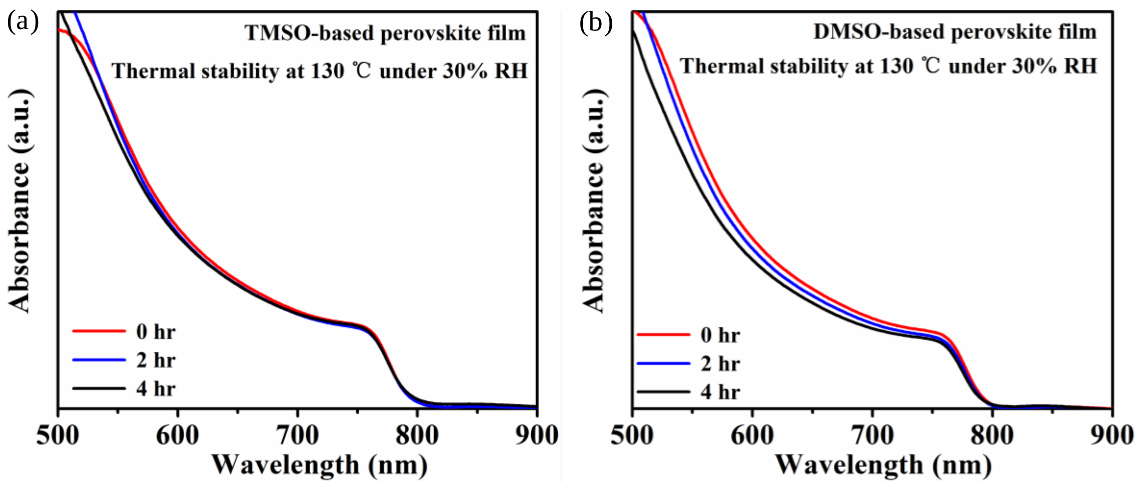

Figure S10. Thermal stability of perovskite film based on different ligand. UV-visible absorption spectra of (a) TMSO- and (b) DMSO-based films annealed at 130 °C for different time under 30% RH.

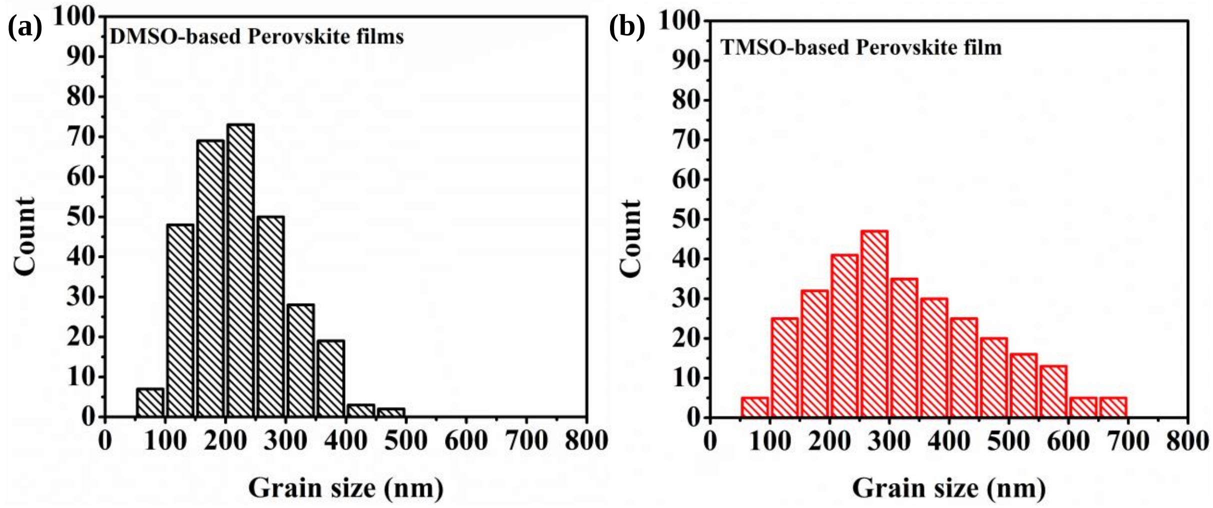

Figure S11. Statistical distribution of the grain size for the (a) DMSO- and (b) TMSO-based perovskite films (number of samples: 300).

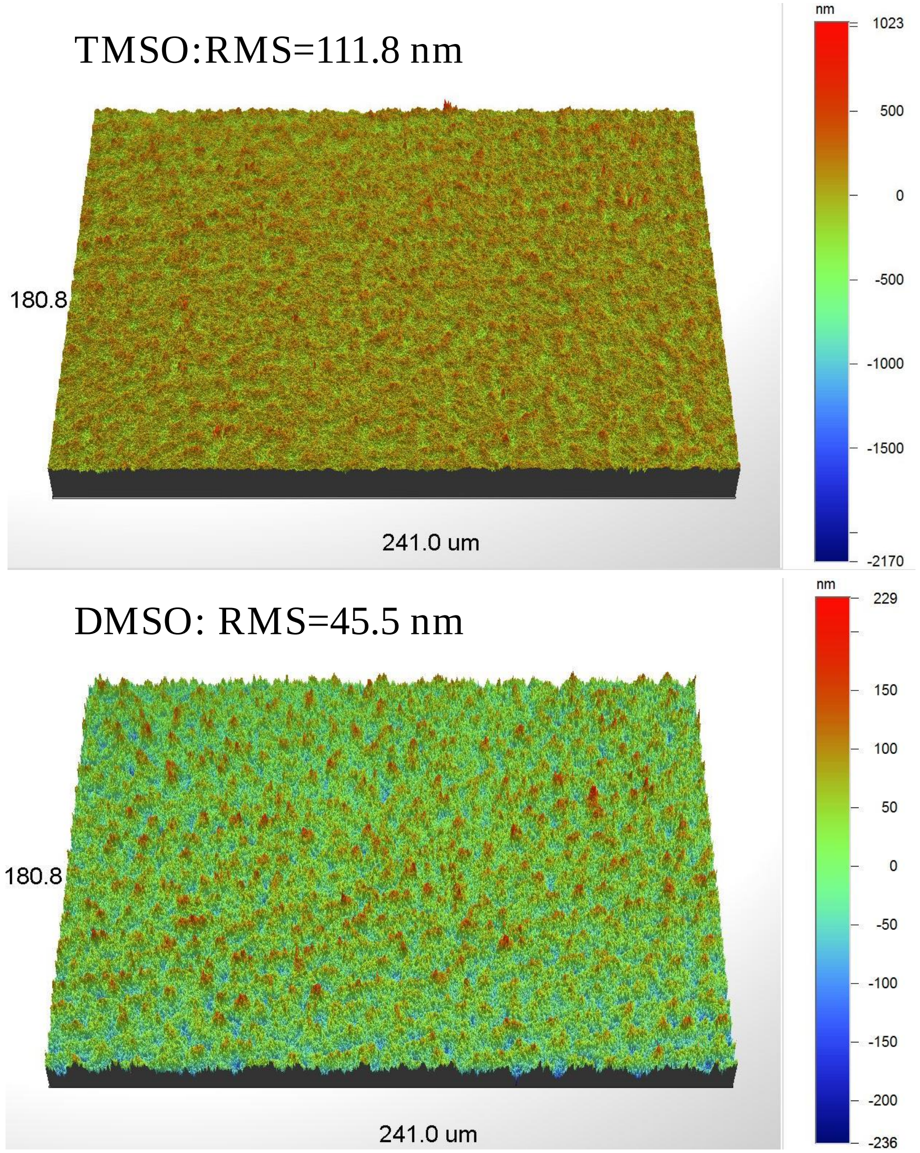

Figure S12. Surface roughness of the TMSO- and DMSO-based annealed perovskite films.

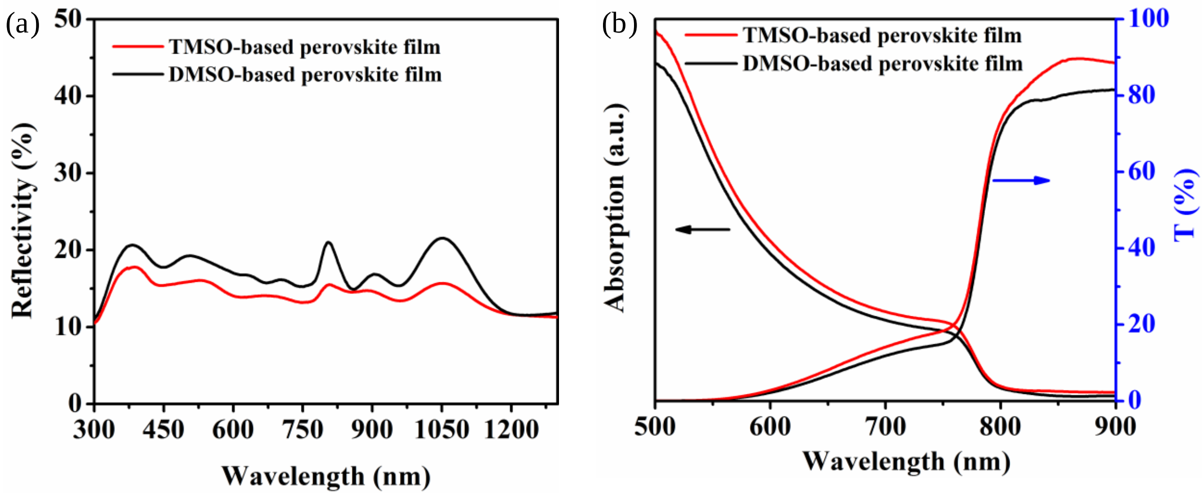

Figure S13. (a) Reflectivity, and (b) absorption, transmittance of the TMSO- and DMSO-based perovskite films. The measurement of reflectivity is performed from film side.

.

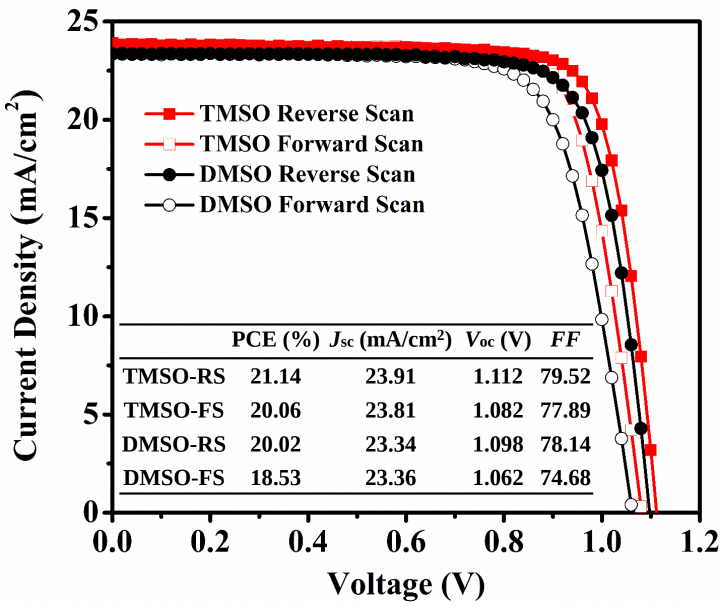

Figure S14. Best device performance of the TMSO- and DMSO-based perovskite solar cells under reverse scan (1.2 V to -0.1 V) and forward scan (-0.1 V to 1.2 V).

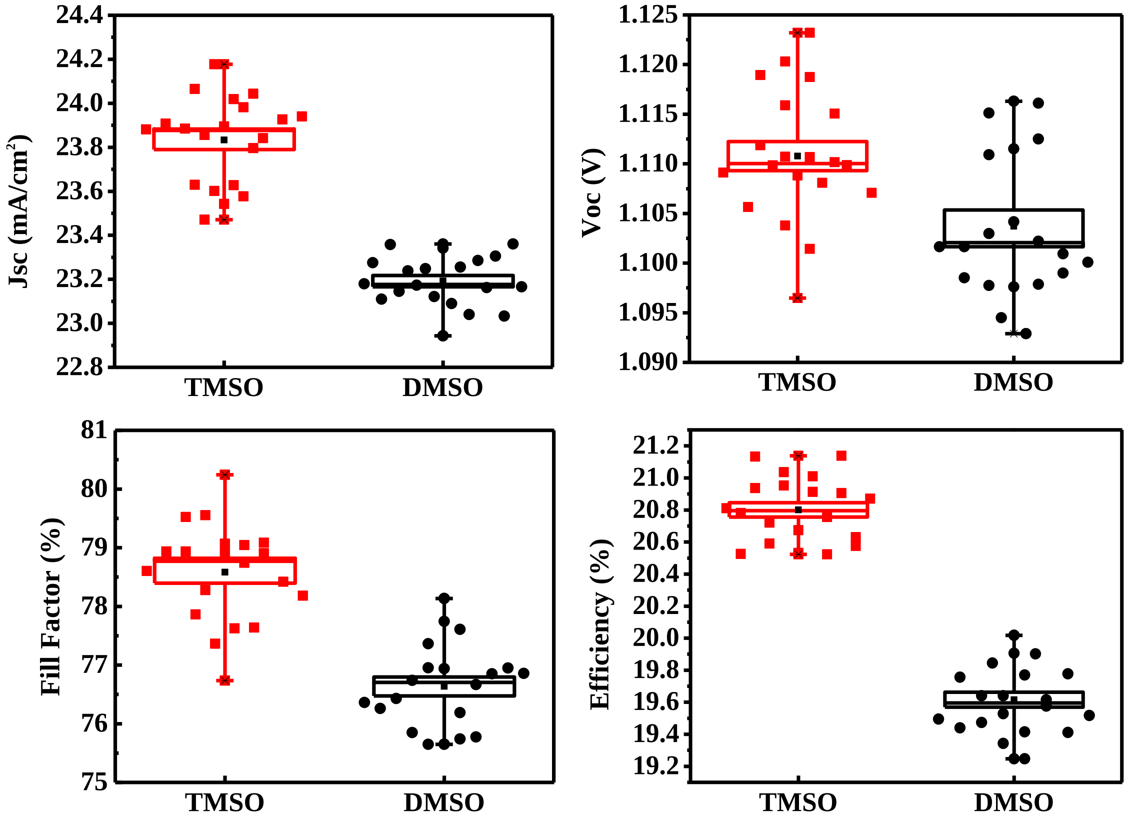

Figure S15. Statistics of the PV parameter of the TMSO- and DMSO-based perovskite solar cells (reverse scan, 1.2 V to -0.1 V).

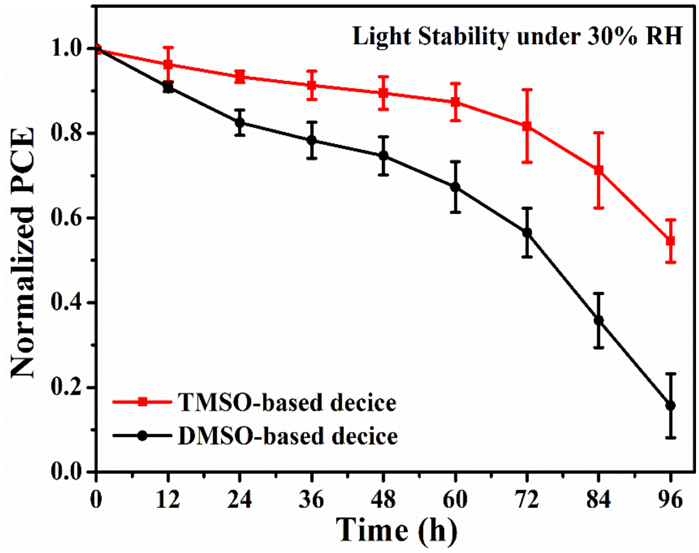

Figure S16. Light stabilities of PSCs based on TMSO and DMSO for 96 hr under 1 sun continuous illumination (30% RH).

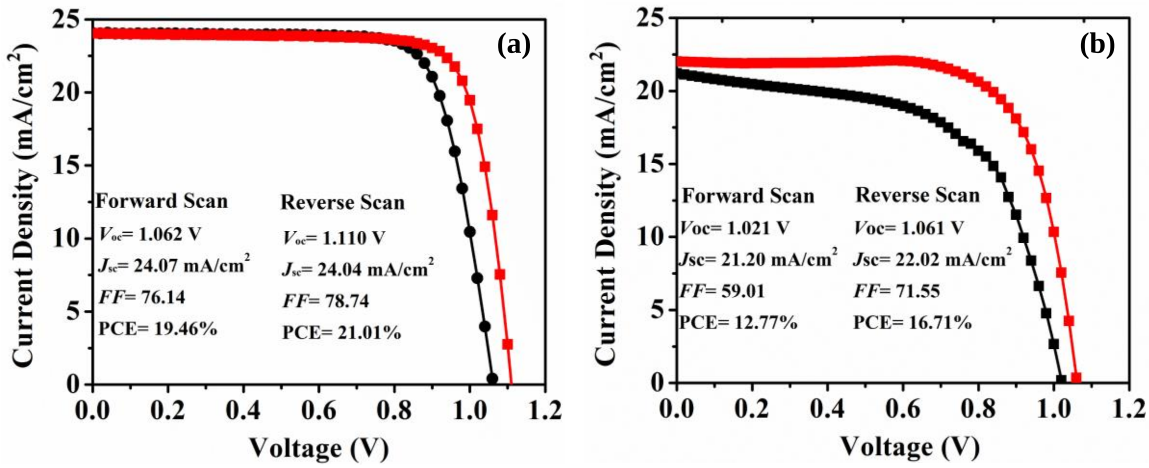

Figure S17. *J-V* curves of the best performing devices of the (a) TMSO- and (b) DMSO-based perovskite solar cells with 20 min storage before annealing under reverse scan (1.2 V to -0.1 V) and forward scan (-0.1 V to 1.2 V). The device area is 0.09 cm^2.
